# Targeting tau for Alzheimers disease through OGA inhibition

**DOI:** 10.1016/j.tjpad.2025.100309

**Published:** 2025-07-24

**Authors:** Michael S. Rafii

**Affiliations:** Alzheimer’s Therapeutic Research Institute, Keck School of Medicine, University of Southern California, USA

The search for effective disease-modifying therapies in Alzheimer’s disease (AD) has long been marked by both scientific ambition and clinical frustration. Recent approvals of anti-amyloid treatments have validated amyloid-beta as a therapeutic target and marked a turning point in the field. At the same time, growing interest has shifted toward tau pathology, which may offer complementary avenues for intervention [1]. Among the more novel approaches is the modulation of tau through post-translational modifications—specifically, O-linked β-N-acetylglucosaminylation (O-GlcNAcylation) [2]. In this context, the first-in-human Phase 1 study of BIIB113, an oral O-GlcNAcase (OGA) inhibitor, represents a significant and encouraging development [3].

Tau pathology, characterized by the accumulation of neurofibrillary tangles (NFTs), is a hallmark of AD and correlates more closely with cognitive decline than amyloid burden [4]. O-GlcNAcylation is a dynamic modification that competes with phosphorylation on tau protein. Preclinical studies have shown that increasing O-GlcNAcylation—by inhibiting the enzyme OGA—can reduce tau phosphorylation and aggregation, potentially mitigating neurodegeneration.

BIIB113 is designed to inhibit OGA, thereby increasing tau O-GlcNAcylation. The rationale is compelling: by tipping the balance away from hyperphosphorylated tau, the compound may slow or prevent the formation of toxic aggregates. This Phase 1 study was designed to evaluate the safety, tolerability, pharmacokinetics (PK), and target engagement of BIIB113 in healthy volunteers.

The study enrolled 72 healthy adult participants between February 2022 and July 2023. It was structured into three parts: a single-ascending dose (SAD) arm, a multiple-ascending dose (MAD) arm, and a target occupancy substudy using PET imaging.

In the SAD arm, participants received a single dose of BIIB113 at 0.5, 3, 15, or 50 mg, or placebo. The MAD arm involved 14 days of once-daily dosing at 15 or 50 mg. The target occupancy substudy used the novel radioligand [^11^C]BIO-1819,578 to assess OGA engagement in the brain following single or repeated doses of 0.5 or 3 mg.

BIIB113 was generally well tolerated across all dosing regimens. Adverse events were mild, and no serious adverse events or dose-limiting toxicities were reported. This is a critical first hurdle for any CNS-active compound, particularly one targeting a ubiquitous enzyme like OGA.

Pharmacokinetically, BIIB113 demonstrated linear behavior across the tested dose range, with a half-life of approximately 30 h—supporting once-daily dosing. Food intake slowed absorption but did not affect overall exposure, and no clinically meaningful differences were observed between elderly and nonelderly participants. These findings suggest a predictable and manageable PK profile, which is essential for future clinical development.

Perhaps the most exciting aspect of the study is the demonstration of robust target engagement. Using PET imaging, the researchers showed that even low doses of BIIB113 (0.5 mg daily) achieved ≥90 % OGA occupancy in the brain for up to 48 h. This is a critical proof-of-mechanism milestone, confirming that the drug reaches its target at clinically feasible doses.

The use of [^11^C]BIO-1819,578 as a radioligand is itself a noteworthy advancement. It provides a non-invasive, quantitative measure of target engagement, enabling dose optimization and de-risking of future efficacy trials.

While these results are encouraging, they must be interpreted within the context of early-phase research. This was a Phase 1 study in healthy volunteers, not patients with AD. The sample size was small, and the duration short. The exclusion of fertile males likely skewed the sex distribution toward mostly females being enrolled in the cohorts. Moreover, the history of OGA inhibitors in AD is mixed. Previous compounds, such as ceperognastat [5], showed promising biomarker effects but failed to demonstrate clinical benefit—and in some cases, worsened cognitive outcomes.

This raises important questions about the therapeutic window and long-term effects of OGA inhibition. OGA is involved in the regulation of many proteins beyond tau, and chronic inhibition could have unintended consequences. The high target occupancy achieved by BIIB113 is impressive, but whether this translates into clinical efficacy—or good tolerability—in patients with AD remains to be seen.

The Phase 1 study of BIIB113 demonstrates that the compound is safe, well tolerated, and achieves high levels of target engagement in the brain. These findings provide a strong rationale for advancing BIIB113 into patient populations, where its true therapeutic potential can be evaluated.

As the field continues to diversify its approach to AD, targeting tau through O-GlcNAcylation represents a promising and mechanistically distinct strategy. BIIB113 may yet prove to be a valuable addition to the therapeutic arsenal—if future trials confirm that its biological effects translate into meaningful clinical outcomes.

## Financial disclosure

MSR has received NIH Grants: R01AG073979; R61AG066543; R33AG066543; U19AG068054; USC Contracts: Eisai and Eli Lilly; Consultant to AC Immune and Ionis; Data Safety Monitoring Board/Scientific Advisory Board for Alzheon, Alnylam, Biohaven, Embic, Positrigo, Recall Therapeutics.

Declaration of Generative AI and AI-assisted technologies in the writing process. No AI was used.

## CRediT authorship contribution statement

**Michael S. Rafii:** Conceptualization, Writing – original draft, Writing – review & editing.

## Declaration of competing interest

The authors declare the following financial interests/personal relationships which may be considered as potential competing interests: Michael Rafii reports a relationship with Eisai Inc that includes: funding grants. Michael Rafii reports a relationship with Eli Lilly and Company that includes: funding grants. Michael Rafii reports a relationship with Alzheon Inc that includes: consulting or advisory. Michael Rafii reports a relationship with Alnylam UK Ltd. that includes: consulting or advisory. Michael Rafii reports a relationship with Ionis Pharmaceuticals Inc that includes: consulting or advisory. Michael Rafii reports a relationship with Embic that includes: consulting or advisory. Michael Rafii reports a relationship with Positrigo that includes: consulting or advisory. Michael Rafii reports a relationship with Recall Therapeutics that includes: consulting or advisory. Michael Rafii reports a relationship with Biohaven Ltd that includes: consulting or advisory. If there are other authors, they declare that they have no known competing financial interests or personal relationships that could have appeared to influence the work reported in this paper.
